# Associations of education and income with hazardous drinking among postpartum women in Japan: results from the TMM BirThree Cohort Study

**DOI:** 10.1186/s12199-021-00991-9

**Published:** 2021-07-03

**Authors:** Keiko Murakami, Mami Ishikuro, Fumihiko Ueno, Aoi Noda, Tomomi Onuma, Fumiko Matsuzaki, Hirohito Metoki, Taku Obara, Shinichi Kuriyama

**Affiliations:** 1 Tohoku Medical Megabank Organization, Tohoku University, 2-1 Seiryo-machi, Aoba-ku, Sendai, Miyagi 980-8573 Japan; 2 Graduate School of Medicine, Tohoku University, 2-1 Seiryo-machi, Aoba-ku, Sendai, Miyagi 980-8575 Japan; 3 Department of Pharmaceutical Sciences, Tohoku University Hospital, 1-1 Seiryo-machi, Aoba-ku, Sendai, Miyagi 980-8574 Japan; 4 Division of Public Health, Hygiene and Epidemiology, Faculty of Medicine, Tohoku Medical and Pharmaceutical University, 1-15-1 Fukumuro, Miyagino-ku, Sendai, Miyagi 983-8536 Japan; 5 Department of Disaster Public Health, International Research Institute of Disaster Science, Tohoku University, 2-1 Seiryo-machi, Aoba-ku, Sendai, Miyagi 980-8573 Japan

**Keywords:** Education, Hazardous drinking, Income, Japan, Postpartum women

## Abstract

**Background:**

Although the postpartum period is suggested to provide an ideal opportunity for interventions to prevent hazardous drinking, evidence on the associations of education and income with hazardous drinking during this period is limited, including in Japan.

**Methods:**

We analyzed data from 11,031 women who participated in the Tohoku Medical Megabank Project Birth and Three-Generation Cohort Study in Japan. Hazardous drinking was defined as ethanol intake of ≥20 g/day 1 year after delivery. We conducted multiple logistic regression analyses to examine whether educational attainment or equivalent household income was associated with hazardous drinking, adjusting for age, parity, drinking status during pregnancy, work status, postpartum depression, breastfeeding, and income/education. We also conducted stratified analyses by income and education groups.

**Results:**

The prevalence of hazardous drinking 1 year after delivery was 3.6%. Lower education was associated with hazardous drinking; the odds ratio (95% confidence interval) of high school education or lower compared with university education or higher was 2.17 (1.59–2.98). Lower income was also associated with hazardous drinking, but this association disappeared after further adjustments for education; the odds ratios (95% confidence intervals) of the lowest compared with highest level of income were 1.42 (1.04–1.94) and 1.12 (0.81–1.54), respectively. A significant interaction was detected; lower education and lower income were associated with increased risks of hazardous drinking only in a lower income group and lower education group, respectively.

**Conclusions:**

Postpartum women with lower education and lower income had higher risks of hazardous drinking in Japan.

**Supplementary Information:**

The online version contains supplementary material available at 10.1186/s12199-021-00991-9.

## Background

Harmful use of alcohol is one of the leading risk factors for disease burden and causes substantial health loss [1]. Approximately 3 million deaths (5.3% of all global deaths) and 132.6 million disability-adjusted life years (5.1% of the global burden of disease and injury) were attributable to alcohol use in 2016 [2]. A hazardous volume of drinking is one of the most important aspects of the risk of alcohol use; a higher amount of alcohol consumed is associated with a higher risk of disease or death [3]. To design appropriate public health policies, it is crucial to identify the population groups that are most affected by hazardous drinking for targeted interventions.

Hazardous drinking is more common among women who are more highly educated and of higher income than among women of lower status in most Organisation for Economic Co-operation and Development (OECD) countries [4]. The patterns of women’s drinking may change over their reproductive life course; most women reduce or abstain from alcohol use once they learn that they are pregnant [5], and many women have been found to return to drinking after their child is born [6, 7]. This suggests that the postpartum period provides an ideal opportunity for interventions to prevent hazardous drinking. Limited evidence on the associations of education and income with hazardous drinking during the postpartum period is inconclusive in Western countries; some studies showed the associations of higher education and higher income with hazardous drinking [6, 8], while others showed no association [9, 10]. To our knowledge, no studies have examined the association of education and income with hazardous drinking among postpartum women in Asia, including Japan, although social and cultural contexts influence different social patterns of drinking in different countries [11]. Studies in Japan should be needed because Japanese women have unique characteristics of drinking. Unlike in other OECD countries, women with lower education in Japan have a higher risk of hazardous drinking [12], and income is not associated with hazardous drinking among women in Japan [12, 13].

Considering the above circumstances, we aimed to examine the associations of education and income with hazardous drinking among postpartum women in Japan. We focused on hazardous drinking 1 year after delivery because there is evidence that parenting a child <1 year of age is associated with reduced alcohol use and that this protective effect diminishes after 1 year postpartum [14].

## Methods

### Study population

**Fig. 1:**
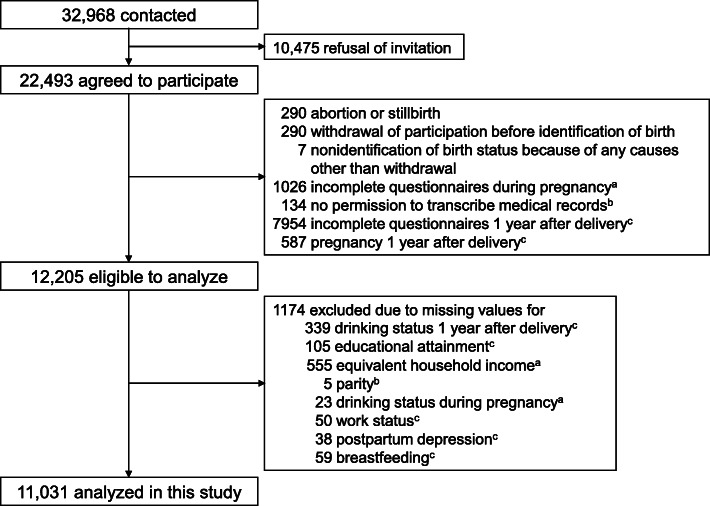
Flow diagram of study participants in the present analysis of the TMM BirThree Cohort Study. ^a^Data on equivalent household income and drinking status during pregnancy were obtained using questionnaires administered during pregnancy. ^b^Data on parity were obtained by transcribing medical records from obstetric clinics or hospitals. ^c^Data on pregnancy status 1 year after delivery, drinking status 1 year after delivery, educational attainment, work status, postpartum depression, and breastfeeding were obtained using questionnaires administered 1 year after delivery

We used data obtained from the Tohoku Medical Megabank Project Birth and Three-Generation Cohort Study (TMM BirThree Cohort Study), which has been described elsewhere [15, 16]. Pregnant women and their family members were contacted in approximately 50 obstetric clinics or hospitals in Miyagi Prefecture when they scheduled their deliveries from 2013 to 2017. Tohoku University Tohoku Medical Megabank Organization established seven community support centers in Miyagi Prefecture as local facilities for voluntary admission-type recruitment and health assessment of the participants [17]. Trained genome medical research coordinators were placed in each clinic, hospital, or community support center to provide information on the TMM BirThree Cohort Study to potential participants and to receive signed informed consent forms from each participant. Of 32,986 pregnant women who were contacted, 22,493 agreed to participate. Among them, 10,288 women were excluded because of abortion or stillbirth, nonidentification of their birth status, incomplete questionnaires, no permission to transcribe their medical records, and pregnancy 1 year after delivery. Of the remaining 12,205 women, 1174 were excluded due to missing values for their drinking status 1 year after delivery, educational attainment, equivalent household income, parity, drinking status during pregnancy, work status, postpartum depression, or breastfeeding. The present study analyzed the remaining 11,031 women (Fig. 1). The Ethics Committee of Tohoku University Tohoku Medical Megabank Organization reviewed and approved the TMM BirThree Cohort Study protocol (2013-1-103-1). The characteristics of the 11,031 analyzed women and the 11,462 excluded women are shown in Table S1.

### Measures

Based on the questionnaire 1 year after delivery, educational attainment was categorized as high school or lower (elementary, junior high school, or senior high school), college (2-year college or special training school), and university or higher (university or graduate school). Using the questionnaire during pregnancy, the participants were asked to select their total annual household income from seven categories: ≤1.99, 2.00–3.99, 4.00–5.99, 6.00–7.99, 8.00–9.99, 10.00–11.99, and ≥12.00 million Japanese yen (JPY). The equivalent household income was calculated as the household income divided by the square root of the number of family members [18]. The resulting value was categorized into four groups approximately corresponding to quartiles: ≤1.99, 2.00–2.99, 3.00–3.99, and ≥4.00 million JPY.

Using the questionnaire 1 year after delivery, the participants were asked to select one of the following response options for their drinking status: current drinker, past drinker, never-drinker, and constitutionally never-drinker. Current drinkers were asked to report the types of alcoholic beverages consumed as well as the frequency and amount of each consumption per day in the past year: *sake* (rice wine), *shochu*, beer, whisky, and/or wine. A score to each category of alcohol consumption frequency was assigned as follows: 7 for every day, 5.5 for 5–6 days/week, 3.5 for 3–4 days/week, 1.5 for 1–2 days/week, 0.5 for a few times/month, and 0 for almost never. The ethanol equivalent intake in grams was calculated as follows: 180 ml *sake* = 23 g, 180 ml *shochu* = 36 g, 633 ml beer = 23 g, 30 ml whisky = 10 g, and 100 ml wine = 12 g [19]. Finally, the weekly ethanol equivalent intake was estimated by multiplying the amount of ethanol by the frequency score; the daily ethanol equivalent intake was calculated by dividing these estimates by 7. Past drinkers, never-drinkers, and constitutionally never-drinkers were considered abstainers and were assigned 0 g/day. The second term of Health Japan 21, which is a 10-year national plan begun in 2013, has established the targets relating to reducing the number of people drinking quantities of alcohol that increase the risk of lifestyle-related disease onset: ethanol intake of ≥40 g/day for men and ≥20 g/day for women [20]. Following this national recommendation, the threshold of hazardous drinking was defined as ethanol intake of ≥20 g/day in the present study. This is in line with the thresholds observed in several countries [4].

As covariates, we chose age, parity, drinking status during pregnancy, work status, postpartum depression, and breastfeeding. Age at delivery and parity were ascertained from the participants’ medical records. Age at delivery was categorized as ≤29, 30–34, and ≥35 years. Parity was dichotomized into nulliparous and multiparous. Using the questionnaire during pregnancy, the participants were asked to select one of the following responses concerning their drinking status: current drinker, past drinker, never-drinker, and constitutionally never-drinker. Responses were dichotomized into drinking (current drinker) and non-drinking (past drinker, never-drinker, and constitutionally never-drinker). Information on the participants’ work status, postpartum depression, and breastfeeding was obtained from the questionnaire 1 year after delivery. The work status was dichotomized as working (permanent worker, self-employed worker, temporary worker, or part-time worker) and not working (on leave, pensioner, housewife, student, or unemployed). The participants provided responses to the Japanese version of the Edinburgh Postpartum Depression Scale, which comprises 10 items assessing any symptoms of depression in the past 7 days [21]. Each item has four possible responses with scores of 0–3, and the total score ranges from 0 to 30. Postpartum depression was defined as a score of ≥9 [22]. All participants were also asked whether their child had been breastfed.

### Statistical analysis

The characteristics of non-drinkers and drinkers 1 year after delivery were compared using the chi-square test. We conducted multiple logistic regression analyses to examine the associations of education and income with hazardous drinking 1 year after delivery. The odds ratios (ORs) and 95% confidence intervals (CIs) were calculated for education and income, adjusted for age (model 1), as well as for parity, drinking status during pregnancy, work status, postpartum depression, and breastfeeding (model 2a for education and model 2b for income). Income and education were then entered simultaneously in the same model (model 3). We also conducted sensitivity analyses restricted to only drinkers 1 year after delivery.

We entered the interaction term between education and income into the model and found that the interaction was statistically significant (*p* < 0.01). Based on this result, we also conducted stratified analyses of the association between education and hazardous drinking by income groups (≥3.00 vs. 0–2.99 million JPY) and the association between income and hazardous drinking by education groups (college or higher vs. high school or lower).

All analyses were conducted using the SAS version 9.4 software (SAS Institute Inc., Cary, NC, USA). For all analyses, a two-tailed *p* value of <0.05 was considered statistically significant.

## Results

Table 1 shows the characteristics of the study participants. About one-third of the participants had graduated from high school or lower. Among the participants, 2405 (21.8%) were drinkers 1 year after delivery. Drinkers were more likely to be less educated, have a lower equivalent household income, be multiparous, be drinkers during pregnancy, and be classified as working and were less likely to breastfeed.

**Table 1:** Characteristics of participants: the Tohoku Medical Megabank Project Birth and Three-Generation Cohort Study

	Total (*N*=11,031)		Drinking status 1 year after delivery				*p* value^a^
			Non-drinkers (*n*=8626)		Drinkers (*n*=2405)		
	n	(%)	n	(%)	n	(%)	
Educational attainment							<0.001
University or higher	3268	(29.6)	2602	(30.2)	666	(27.7)	
College	4263	(38.7)	3367	(39.0)	896	(37.3)	
High school or lower	3500	(31.7)	2657	(30.8)	843	(35.0)	
Equivalent household income							0.0073
≥4.00 million JPY	2909	(26.4)	2271	(26.3)	638	(26.5)	
3.00–3.99 million JPY	2068	(18.7)	1674	(19.4)	394	(16.4)	
2.00–2.99 million JPY	3514	(31.9)	2712	(31.5)	802	(33.4)	
≤1.99 million JPY	2540	(23.0)	1969	(22.8)	571	(23.7)	
Covariates							
Age							0.13
≤29 years	3226	(29.2)	2512	(29.1)	714	(29.7)	
30–34 years	4211	(38.2)	3334	(38.7)	877	(36.5)	
≥35 years	3594	(32.6)	2780	(32.2)	814	(33.9)	
Multiparous	5761	(52.2)	4327	(50.2)	1434	(59.6)	<0.001
Drinking during pregnancy	701	(6.4)	357	(4.1)	344	(14.3)	<0.001
Working	5815	(52.7)	4382	(50.8)	1433	(59.6)	<0.001
Postpartum depression	1423	(12.9)	1114	(12.9)	309	(12.9)	0.93
Breastfeeding	6741	(61.1)	6053	(70.2)	688	(28.6)	<0.001

*JPY*, Japanese yen

^a^Obtained using the chi-square test, comparing non-drinkers and drinkers

Table 2 shows the prevalence, ORs, and 95% CIs for hazardous drinking 1 year after delivery. The prevalence of hazardous drinking was 3.6%. Lower educational attainment was associated with an increased risk of hazardous drinking after adjusting for age (model 1) and after additionally adjusting for parity, drinking status during pregnancy, work status, postpartum depression, and breastfeeding (model 2a); the adjusted ORs of high school education or lower compared with university education or higher were 3.01 (95% CI, 2.25–4.04) and 2.26 (95% CI, 1.67–3.06), respectively. This association was somewhat attenuated but did not disappear after further adjustments for equivalent household income (model 3); the corresponding OR was 2.17 (95% CI, 1.59–2.98). Lower equivalent household income was associated with an increased risk of hazardous drinking after adjusting for age (model 1) and after additionally adjusting for parity, drinking status during pregnancy, work status, postpartum depression, and breastfeeding (model 2b); the adjusted ORs of ≤1.99 million JPY compared with ≥4.00 million JPY income were 1.87 (95% CI, 1.40–2.49) and 1.42 (95% CI, 1.04–1.94), respectively. This association disappeared after further adjustments for education (model 3); the corresponding OR was 1.12 (95% CI, 0.81–1.54). Older age was associated with an increased risk of hazardous drinking after multivariate adjustments. Multiparity, drinking during pregnancy, and non-breastfeeding were associated with increased risks of hazardous drinking, while work status and postpartum depression were not associated with hazardous drinking. Sensitivity analyses restricted to only drinkers 1 year after delivery produced similar results (Table S2).

**Table 2:** Associations of education and income with hazardous drinking 1 year after delivery

	Hazardous drinking/participants	(%)	Model 1	Model 2a	Model 2b	Model 3
			OR (95% CI)	OR (95% CI)	OR (95% CI)	OR (95% CI)
Total	397/11031	(3.6)				
Educational attainment						
University or higher	62/3268	(1.9)	1.00	1.00		1.00
College	150/4263	(3.5)	1.89 (1.40–2.55)	1.65 (1.22–2.24)		1.62 (1.19–2.22)
High school or lower	185/3500	(5.3)	3.01 (2.25–4.04)	2.26 (1.67–3.06)		2.17 (1.59–2.98)
Equivalent household income						
≥4.00 million JPY	83/2909	(2.9)	1.00		1.00	1.00
3.00–3.99 million JPY	46/2068	(2.2)	0.80 (0.56–1.16)		0.89 (0.61–1.30)	0.80 (0.55–1.17)
2.00–2.99 million JPY	143/3514	(4.1)	1.51 (1.15–1.99)		1.37 (1.03–1.83)	1.18 (0.88–1.58)
≤1.99 million JPY	125/2540	(4.9)	1.87 (1.40–2.49)		1.42 (1.04–1.94)	1.12 (0.81–1.54)
Covariates						
Age						
≤29 years	101/3226	(3.1)	1.00	1.00	1.00	1.00
30–34 years	153/4211	(3.6)	1.17 (0.90–1.51)	1.34 (1.03–1.76)	1.28 (0.98–1.67)	1.37 (1.05–1.79)
≥35 years	143/3594	(4.0)	1.28 (0.99–1.66)	1.43 (1.09–1.88)	1.41 (1.07–1.86)	1.47 (1.12–1.95)
Parity						
Nulliparous	128/5270	(2.4)	1.00	1.00	1.00	1.00
Multiparous	269/5761	(4.7)	1.94 (1.56–2.41)	1.90 (1.52–2.37)	1.81 (1.42–2.29)	1.76 (1.39–2.24)
Drinking status during pregnancy						
Non-drinking	328/10330	(3.2)	1.00	1.00	1.00	1.00
Drinking	69/701	(9.8)	3.30 (2.52–4.34)	3.15 (2.37–4.19)	3.13 (2.36–4.17)	3.15 (2.37–4.19)
Work status						
Not working	161/5216	(3.1)	1.00	1.00	1.00	1.00
Working	236/5815	(4.1)	1.33 (1.08–1.63)	1.16 (0.94–1.43)	1.14 (0.92–1.40)	1.17 (0.95–1.45)
Postpartum depression						
No	334/9608	(3.5)	1.00	1.00	1.00	1.00
Yes	63/1423	(4.4)	1.32 (1.00–1.74)	1.22 (0.92–1.62)	1.21 (0.91–1.61)	1.20 (0.90–1.59)
Breastfeeding						
Yes	78/6741	(1.2)	1.00	1.00	1.00	1.00
No	319/4290	(7.4)	7.05 (5.48–9.07)	6.75 (5.23–8.71)	7.07 (5.48–9.11)	6.72 (5.21–8.67)

*CI*, confidence interval; *JPY*, Japanese yen; *OR*, odds ratio

Model 1: adjusted for age (for all variables in the table)

Model 2a (for education) and 2b (for income): model 1 + adjusted for parity, drinking status during pregnancy, work status, postpartum depression, and breastfeeding

Model 3: model 2 + adjusted for equivalent household income/educational attainment

Table 3 presents the prevalence, ORs, and 95% CIs for hazardous drinking 1 year after delivery according to income groups. Lower educational attainment was associated with an increased risk of hazardous drinking not in a higher income group (≥3.00 million JPY) but in a lower income group (0–2.99 million JPY); the adjusted ORs of high school education or lower compared with university education or higher were 1.06 (95% CI, 0.64–1.75) and 3.83 (95% CI, 2.29–6.40), respectively (interaction *p* < 0.001).

**Table 3:** Associations between education and hazardous drinking 1 year after delivery according to income groups

	Hazardous drinking/participants	(%)	Age-adjusted	Multivariate-adjusted^a^
			OR (95% CI)	OR (95% CI)
Educational attainment				
Higher income group (*n*=4977)				
University or higher	45/2082	(2.2)	1.00	1.00
College	58/1938	(3.0)	1.39 (0.94–2.07)	1.27 (0.85–1.90)
High school or lower	26/957	(2.7)	1.27 (0.78–2.07)	1.06 (0.64–1.75)
Lower income group (*n*=6054)				
University or higher	17/1186	(1.4)	1.00	1.00
College	92/2325	(4.0)	2.84 (1.69–4.79)	2.54 (1.50–4.31)
High school or lower	159/2543	(6.3)	4.87 (2.94–8.09)	3.83 (2.29–6.40)

*CI*, confidence interval; *OR*, odds ratio

Interaction between income groups and educational attainment: *p*<0.001. Equivalent household income was dichotomized into higher (≥3.00 million Japanese yen) and lower (0–2.99 million Japanese yen) groups

^a^Adjusted for age, parity, drinking status during pregnancy, work status, postpartum depression, and breastfeeding

Table 4 presents the prevalence, ORs, and 95% CIs for hazardous drinking 1 year after delivery according to education groups. Lower equivalent household income was associated with an increased risk of hazardous drinking not in a higher education group (college or higher) but in a lower education group (high school or lower); the adjusted ORs of ≤1.99 million JPY compared with ≥4.00 million JPY income were 0.99 (95% CI, 0.64–1.53) and 2.45 (95% CI, 1.23–4.86), respectively (interaction *p* = 0.0085).

**Table 4:** Associations between income and hazardous drinking 1 year after delivery according to education groups

	Hazardous drinking/participants	(%)	Age-adjusted	Multivariate-adjusted^a^
			OR (95% CI)	OR (95% CI)
Equivalent household income				
Higher education group (*n*=7531)				
≥4.00 million JPY	73/2449	(3.0)	1.00	1.00
3.00–3.99 million JPY	30/1571	(1.9)	0.67 (0.44–1.04)	0.79 (0.50–1.23)
2.00–2.99 million JPY	71/2278	(3.1)	1.13 (0.81–1.57)	1.08 (0.76–1.53)
≤1.99 million JPY	38/1233	(3.1)	1.12 (0.75–1.67)	0.99 (0.64–1.53)
Lower education group (*n*=3500)				
≥4.00 million JPY	10/460	(2.2)	1.00	1.00
3.00–3.99 million JPY	16/497	(3.2)	1.53 (0.69–3.41)	1.59 (0.70–3.59)
2.00–2.99 million JPY	72/1236	(5.8)	2.85 (1.46–5.59)	2.60 (1.31–5.16)
≤1.99 million JPY	87/1307	(6.7)	3.32 (1.70–6.46)	2.45 (1.23–4.86)

*CI*, confidence interval; *JPY*, Japanese yen; *OR*, odds ratio

Interaction between education groups and equivalent household income: *p*=0.0085. Educational attainment was dichotomized into higher (college or higher) and lower (high school or lower) groups

^a^Adjusted for age, parity, drinking status during pregnancy, work status, postpartum depression, and breastfeeding

## Discussion

The present study was performed to examine the associations of education and income with hazardous drinking among postpartum women in Japan. The prevalence of hazardous drinking 1 year after delivery was 3.6%. Lower education was associated with an increased risk of hazardous drinking. Lower income was also associated with an increased risk of hazardous drinking, but this association disappeared after further adjustments for education. A significant interaction was detected between education and income; lower education and lower income were associated with increased risks of hazardous drinking only in a lower income group and lower education group, respectively.

The prevalence of hazardous drinking among women 1 year after delivery was 3.6%. We could not directly compare the prevalence of hazardous drinking 1 year after delivery with corresponding data in Japan because there is little information on hazardous drinking among postpartum women. The National Health and Nutrition Survey in Japan reported that women who were hazardous drinkers comprised about 7% in their twenties (5.9% in 2014, 8.1% in 2015, 7.3% in 2016, and 5.5% in 2017) and about 11% in their thirties (12.8% in 2014, 9.3% in 2015, 10.1% in 2016, and 11.3% in 2017) [13]. The 2013 national survey among the general population of Japan revealed that the prevalence of hazardous drinking was 4.5% among women in their twenties and 10.8% among women in their thirties [12]. Taken together, it is assumed that the prevalence of hazardous drinking 1 year after delivery observed in the present study was lower than that among women in the general population. There are at least two possible explanations for this lower prevalence. One is that reductions in women’s drinking are the most pronounced during the first year postpartum, and the level of alcohol use subsequently increases with the age of the child [14]. The other is that motherhood is associated with decreased drinking overall; women with small children consume less alcohol than women without children [14, 23]. Motherhood represents a significant change in a woman’s life and involves increased responsibilities and expectations. There is some evidence that transitions into traditional adult roles such as marriage and parenthood are associated with reductions in excessive drinking [24]. Being the mother of a small child may also restrict a woman’s opportunities to go out for dinner or a party or to take part in other social activities in which she could drink excessively [25]. This new role and change in lifestyle may be protective against hazardous drinking. Compared with data on maternal drinking during pregnancy, limited epidemiological data are available on alcohol use during the postpartum period. The present study can contribute to the limited evidence on the prevalence of hazardous drinking during the postpartum period because it provided reliable estimates crucial for designing appropriately targeted prevention and early intervention initiatives.

Women with lower education were more likely to be hazardous drinkers. This result is inconsistent with the findings in Western countries, in which an association has been found between higher education and hazardous drinking among women in the general population [4] and among postpartum women [6, 8]. The 2013 national survey among the general population of Japan showed that lower-educated women were more likely to be hazardous drinkers [12], which is consistent with the present finding. There are at least three possible explanations for this association. First, education conveys factual health-related knowledge and raises cognitive skills which affect health-promoting decisions [26]. The National Health and Nutrition Survey 2015 in Japan reported that only 23.6% of women know the amount of alcohol that increases the risk of lifestyle-related disease onset for women [13]. Education may increase an understanding of the negative impacts of hazardous drinking and may build the capacity to manage drinking by stopping or keeping consumption low. Second, education shapes cultural capital in the form of health-related values and norms [27]. Because alcohol drinking is influenced by cultural norms [4], unequal distribution of cultural capital across educational levels may result in differences in alcohol drinking patterns in the social and cultural contexts in Japan [11]. Third, social networks, which combine an individual’s resources with those of others [28], may partially explain the observed association. People with lower education associate with others who also have lower education, and their social networks communicate health-related behaviors [26]. Men with lower education are more likely to be hazardous drinkers in Japan [12, 29], and drinking patterns can follow social networking paths [30]. These situations would lead to the association between lower education and hazardous drinking observed in the present study.

The association between lower income and hazardous drinking disappeared after adjusting for education. This result is inconsistent with the findings in Western countries, in which an association has been found between higher income and hazardous drinking among women in the general population [4] and among postpartum women [6, 8]. The National Health and Nutrition Survey 2014 and the 2013 national survey in the general population of Japan showed no association between income and hazardous drinking among women [12, 13], which is consistent with the present finding. In Western countries, the association between higher income and hazardous drinking is often explained by the fact that affluent people have more disposable income with which to purchase alcohol [31]. However, safe and high-quality varieties of alcoholic beverage are available at relatively low prices in Japan [32], which would partially explain the lack of an association in the present study. This indicates that taxation and pricing policies may be unsuccessful in reducing hazardous drinking among postpartum women in Japan, although these policies have potential to reduce alcohol consumption among hazardous drinkers with lower income in Western countries [33, 34]. The present study also showed that lower income was associated with an increased risk of hazardous drinking among lower-educated women. This finding suggests that the cumulative disadvantage of lower education and lower income would increase the risk of hazardous drinking. Therefore, more attention to these populations may be needed.

The present findings have implications for the design of effective interventions aimed at preventing hazardous drinking among postpartum women. Although most women reduce or abstain from alcohol use during pregnancy [5], 3.6% had already engaged in hazardous drinking 1 year after delivery in the present study. Hazardous drinking among postpartum women is of significant public health concern because maternal drinking can directly and indirectly damage mother and children’s health even at a non-dependent level; it can negatively have impacts on child-rearing environments, increase physical and psychological harm to children, damage mother–child relationships [35], and increase the risk of alcohol-related problems later in life [36]. The second term of Health Japan 21 aims to reduce the prevalence of hazardous drinking among women from 7.5% in 2010 to 6.4% by 2022 [20]. However, this prevalence has been increasing; the prevalence was 9.1% in 2019 [13]. The first year of motherhood is the year of greatest transition and may therefore be associated with changes in drinking patterns [14], which suggests that this period provides an ideal opportunity for primary care providers to intervene. While much attention has been paid to drinking during pregnancy, much less research has focused on mothers’ postpartum drinking. Although one meta-analysis demonstrated the effectiveness of psychosocial interventions to reduce alcohol consumption among mothers with dependent children, both the number of studies included and the effect found was small [37]. The present study has clarified factors that can be used to identify high-risk subpopulations of postpartum women and that can be targeted in future interventions in Japan.

Limitations of the present study should be noted. First, it was conducted in 1 of the 47 prefectures in Japan, which limits the generalizability of the findings. However, the prevalence of hazardous drinking among women in Miyagi Prefecture (8.0% in 2016) [38] was similar to that reported from the National Health and Nutrition Survey (9.1% in 2016) [13]. Second, approximately half of the women who agreed to participate in the TMM BirThree Cohort Study were analyzed. Women who were excluded from the analysis were less educated, had lower equivalent income, and were more likely to engage in hazardous drinking 1 year after delivery (Table S1); this may have led to underestimation of the association of education and income with hazardous drinking. Third, information on equivalent household income was obtained during pregnancy, and it is possible that the women’s income changed between the pregnancy periods and 1 year after delivery. Fourth, as in most epidemiological studies, the participants’ drinking status was self-reported. Women may be influenced by social desirability, a bias that is important especially when questions address socially undesirable attitudes and behaviors. However, one review concluded that a self-report method is a reliable and valid approach to measuring alcohol consumption [39]. Additionally, one study showed that correction of self-reporting bias did not change the direction of educational differences in hazardous drinking among Japanese women [40]. Finally, because of data limitations, we could not examine the associations of education and income with heavy episodic drinking (defined as ethanol intake of ≥60 g on at least one occasion at least once per month) [2]. These associations would differ from those observed in the present study because heavy episodic drinking is different from other forms of drinking such as hazardous drinking, in that the intention is to drink a harmful amount of alcohol [4].

## Conclusion

The present study showed that the prevalence of hazardous drinking 1 year after delivery was 3.6%. Lower education was associated with an increased risk of hazardous drinking. Although the association between lower income and hazardous drinking disappeared after adjusting for education, this association remained only in a lower education group. Our findings can provide clues to designing effective interventions for preventing hazardous drinking among postpartum women, which would improve maternal and child health.

## Supplementary Information

**Additional file 1: Table S1.** Characteristics differences between 11,031 participants who were analyzed and 11,462 participants who were excluded from the analysis **Table S2.** Associations of education and income with hazardous drinking 1 year after delivery among drinkers

## Data Availability

The data obtained through the TMM BirThree Cohort Study are incorporated into the TMM biobank. All data analyzed during the present study are available for research purpose with the approval by the Sample and Data Access Committee of the Biobank.

## Abbreviations

95% CI: 95% confidence interval
OR: Odds ratio
TMM BirThree Cohort Study: Tohoku Medical Megabank Project Birth and Three-Generation Cohort Study
