# Supplementary material for: Associations of education and income with hazardous drinking among postpartum women in Japan: results from the TMM BirThree Cohort Study
Source: Environ Health Prev Med. 2021 Jul 3;26:70. doi: 10.1186/s12199-021-00991-9 (PMC8254918; doi:10.1186/s12199-021-00991-9)
Supplement: Supplementary file 1 — Additional file 1: Table S1. Characteristics differences between 11,031 participants who were analyzed and 11,462 participants who were excluded from the analysis Table S2. Associations of education and income with hazardous drinking 1 year after delivery among drinkers [file 12199_2021_991_MOESM1_ESM.pdf]

Supplementary material

Table S1. Characteristics differences between 11,031 participants who were analyzed and 11,462 participants who were excluded from the analysis

	Participants who were analyzed (n=11,031)		Participants who were not analyzed (n=11,462)		*p*-value[a]
	n	(%)	n	(%)	
Educational attainment					<0.001
University or higher	3268	(29.6)	574	(22.8)	
College	4263	(38.7)	996	(39.5)	
High school or lower	3500	(31.7)	952	(37.7)	
Equivalent household income					<0.001
≥4.00 million JPY	2909	(26.4)	1962	(22.6)	
3.00–3.99 million JPY	2068	(18.7)	1521	(17.6)	
2.00–2.99 million JPY	3514	(31.9)	2784	(32.2)	
≤1.99 million JPY	2540	(23.0)	2386	(27.6)	
Covariates					
Age					<0.001
≤29 years	3226	(29.2)	4161	(38.3)	
30–34 years	4211	(38.2)	3775	(34.8)	
≥35 years	3594	(32.6)	2927	(26.9)	
Multiparous	5761	(52.2)	5610	(50.4)	0.0073
Drinking during pregnancy	701	(6.4)	579	(5.9)	0.15
Working	5815	(52.7)	1286	(52.6)	0.95
Postpartum depression	1423	(12.9)	308	(14.8)	0.020
Breastfeeding	6741	(61.1)	1253	(44.6)	<0.001
Hazardous drinking 1 year after delivery	397	(3.6)	110	(4.9)	0.0026

JPY, Japanese yen.

Percentages are shown after excluding participants whose values were missing.

[a]Obtained using the chi-square test, comparing participants who were analyzed and participants who were not.

Table S2. Associations of education and income with hazardous drinking 1 year after delivery among drinkers

	Hazardous drinking /drinkers	(%)	Model 1 OR (95% CI)		Model 2a OR (95% CI)		Model 2b OR (95% CI)		Model 3 OR (95% CI)	
Total	397/2405	(16.5)								
Educational attainment										
University or higher	62/666	(9.3)	1.00		1.00				1.00	
College	150/896	(16.7)	1.97	(1.43–2.69)	1.90	(1.38–2.61)			1.82	(1.32–2.52)
High school or lower	185/843	(22.0)	2.90	(2.12–3.96)	2.67	(1.94–3.67)			2.45	(1.76–3.42)
Equivalent household income										
≥4.00 million JPY	83/638	(13.0)	1.00				1.00		1.00	
3.00–3.99 million JPY	46/394	(11.7)	0.93	(0.63–1.36)			0.97	(0.66–1.44)	0.84	(0.57–1.26)
2.00–2.99 million JPY	143/802	(17.8)	1.52	(1.13–2.05)			1.46	(1.08–1.98)	1.21	(0.89–1.65)
≤1.99 million JPY	125/571	(21.9)	2.01	(1.48–2.74)			1.81	(1.30–2.52)	1.36	(0.96–1.91)
Covariates										
Age										
≤29 years	101/714	(14.2)	1.00		1.00		1.00		1.00	
30–34 years	153/877	(17.5)	1.28	(0.98–1.69)	1.47	(1.10–1.95)	1.42	(1.07–1.89)	1.52	(1.14–2.03)
≥35 years	143/814	(17.6)	1.29	(0.98–1.71)	1.49	(1.11–2.00)	1.49	(1.11–2.01)	1.58	(1.17–2.13)
Parity										
Nulliparous	128/971	(13.2)	1.00		1.00		1.00		1.00	
Multiparous	269/1434	(18.8)	1.48	(1.17–1.86)	1.42	(1.12–1.80)	1.31	(1.02–1.69)	1.27	(0.98–1.64)
Drinking status during pregnancy										
Non-drinking	328/2061	(15.9)	1.00		1.00		1.00		1.00	
Drinking	69/344	(20.1)	1.30	(0.98–1.74)	1.38	(1.02–1.86)	1.38	(1.03–1.86)	1.37	(1.01–1.84)
Work status										
Not working	161/972	(16.6)	1.00		1.00		1.00		1.00	
Working	236/1433	(16.5)	0.99	(0.80–1.24)	1.06	(0.85–1.33)	1.04	(0.83–1.30)	1.09	(0.86–1.37)
Postpartum depression										
No	334/2096	(15.9)	1.00		1.00		1.00		1.00	
Yes	63/309	(20.4)	1.41	(1.04–1.90)	1.30	(0.95–1.77)	1.29	(0.95–1.76)	1.25	(0.92–1.71)
Breastfeeding										
Yes	78/688	(11.3)	1.00		1.00		1.00		1.00	
No	319/1717	(18.6)	1.87	(1.43–2.45)	2.00	(1.52–2.63)	2.03	(1.54–2.67)	1.98	(1.50–2.61)

CI, confidence interval; JPY, Japanese yen; OR, odds ratio.
Model 1: adjusted for age (for all variables in the table).

Model 2a (for education) and 2b (for income): model 1 + adjusted for parity, drinking status during pregnancy, work status, postpartum depression, and breastfeeding.
Model 3: model 2 + adjusted for equivalent household income/educational attainment.
